# New hits as phase II enzymes inducers from a focused library with heteroatom–heteroatom and Michael-acceptor motives

**DOI:** 10.4155/fso.15.18

**Published:** 2015-11-01

**Authors:** Mauricio Cabrera, Stefani de Ovalle, Mariela Bollati-Fogolín, Fabiana Nascimento, Patrícia Corbelini, Fernanda Janarelli, Daniel Kawano, Vera Lucia Eifler-Lima, Mercedes González, Hugo Cerecetto

**Affiliations:** 1 Grupo de Química Medicinal, Laboratorio de Química Orgánica, Facultad de Ciencias-Facultad de Química, Universidad de la República, 11400 Montevideo, Uruguay; 2 Cell Biology Unit, Institut Pasteur Montevideo, 11400 Montevideo, Uruguay; 3 Laboratório de Síntese Orgânica Medicinal/LaSOM, Faculdade de Farmácia, Universidade Federal do Rio Grande do Sul/UFRGS, Porto Alegre/RS, Brazil

**Keywords:** 1,2,5-oxadiazol 2-oxide, 1,2,4-triazine 4-oxide, chemopreventive agents, phase II enzyme inducers, tetrahydropyrimidinone

## Abstract

The increased activity of phase-II-detoxification enzymes, such as quinone reductase (QR) and glutation *S*-transferase (GST), correlates with protection against chemically induced carcinogenesis. Herein we studied 11 different chemotypes, pyrazole, 1,2,4-oxadiazole, 1,2,5-oxadiazole, 1,2,3-thiadiazole, 1,2,4-thiazole, 1,3,4-oxathiazole, thienyl hydrazone, α,β-unsaturated-oxime, α,β-unsaturated-*N*-oxide, coumarin and α,β-unsaturated-carbonyl, as phase-II enzymes inducers in order to identify new pharmacophores with chemopreventive activity. Fifty-four compounds were analyzed on wild-type mouse-hepatoma Hepa-1c1c7 and on the aryl-hydrocarbon-nuclear-translocator (Arnt)-defective mutant BpRc1 cells. New monofunctional inducers of QR and GST were identified, the 1,2,5-oxadiazol-2-oxide **(3)**, the 1,2,4-triazine-4-oxides **(23)** and **(32)** and the tetrahydropyrimidinones **(28)** and **(49)**. It was confirmed that Nrf2 nuclear translocation is the operative molecular mechanism that allows compound **(3)** to exert protective effects via expression of downstream phase-II enzymes.

## Abstract (lay abstract)

Cancer chemoprevention is the prevention, delay or reversal of the carcinogenesis by administration of drugs. A group of chemopreventative agents includes quinone reductase and glutation *S*-transferase. Herein we have studied 11 chemotypes, trying to identify new pharmacophores for chemopreventives. We found new inducers of quinone reductase and glutation *S*-transferase, with excellent *in vitro* chemopreventive indexes, the 1,2,5-oxadiazol-2-oxide **(3)**, the 1,2,4-triazine-4-oxides **(23)** and **(32)** and the tetrahydropyrimidinones **(28)** and **(49)**, confirming that Nrf2 nuclear translocation is the operative molecular mechanism that allows compound **(3)** to exert protection. We have therefore highlighted good candidates for further *in vivo* studies of cancer chemopreventive activity.

**Figure 1.**
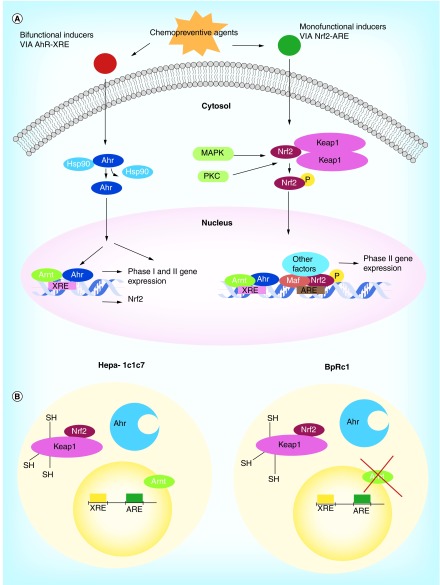
**Biology of the chemoprevention.** **(A)** Model of the relationship between the mechanism of action of monofunctional and bifunctional inducers of phase II enzymes and cancer chemopreventive agents. **(B)** Schemes of the cellular systems employed herein the wild-type Hepa-1c1c7 and the mutant BpRc1 cells.

**Figure 2.**
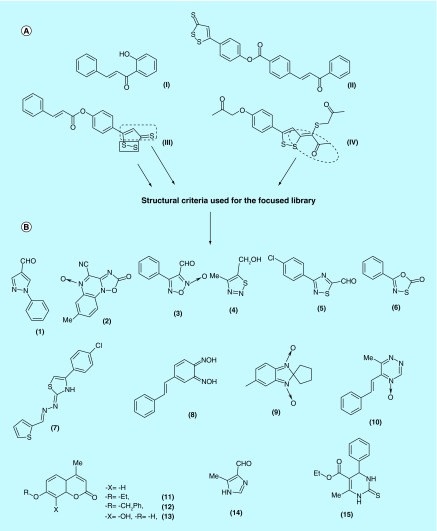
**Structural features used in the focused library.** **(A)** Some previous described cancer chemopreventive agents and structural features used to design the focused library. **(B)** Compounds from our chemical library that meet to the pre-established structural requirements.

**Figure 3.**
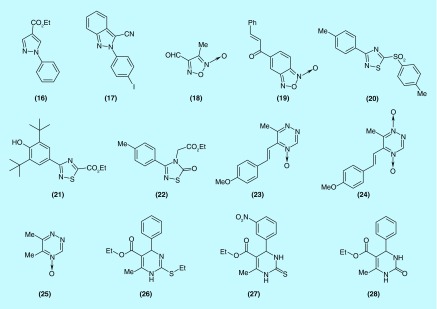
**Second series of studied compounds belonging to pyrazole, 1,2,5-oxadiazole, 1,2,4-thiadiazol, 1,2,4-triazine 4-oxide and tetrahydropyrimidine chemotypes.**

**Figure 4.**
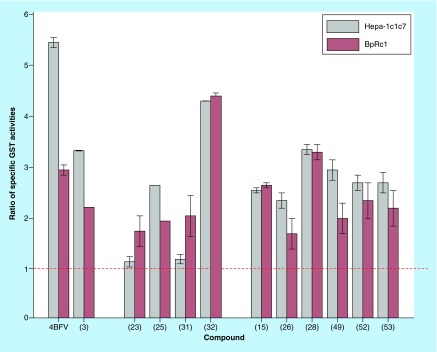
**Induction of glutation *S*-transferase activity by the compounds, at 10 μM dose, on studied cellular models expressed as the ratio of glutation *S*-transferase specific activity.** Ratio of GST specific activity = GST specific activity of treated cells/GST specific activity of control cells. GST specific activity of control cells: for Hepa-1c1c7 line = 0.1300 ±0.0081 U/mg protein, for BpRc1 line = 0.0650 ±0.0010 U/mg protein. Horizontal line dotted indicates the cut of activity. GST: Glutation *S*-transferase.

**Figure 5.**
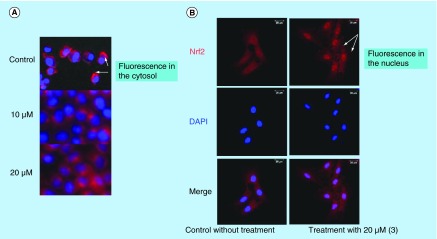
**Subcellular location of Nrf2.** Immunofluorescent staining showing that 1,2,5-oxadiazole (3) increased the localization of Nrf2 (red) in nucleus (blue) in Hepa-1c1c7 cell after treatment. **(A)** Merge of the staining without, or with 10 and 20 μM of compound (3). **(B)** Study using 20 mM of compound (3). Left: untreated cells; Right: treated cells with 20 μM of compound (3).

**Figure 6.**
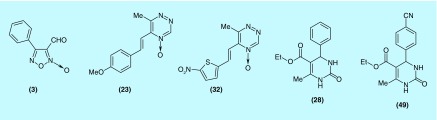
**Some of the new greater monofunctional inducers of both phaseII enzymes, quinone reductase and glutation *S*-transferase, identified herein.**

The process of carcinogenesis involves intricate and prolonged events launched by the cellular biomolecule damages promoted by endogenous or exogenous agents. The prevention of carcinogenesis, cancer chemoprevention, implies the avoidance, the slowing or reversing of the carcinogenic process through the use of drugs named as chemopreventive agents (ChAs). ChAs could act by suppressing the activation by metabolism of the carcinogens or blocking their formation [1]. ChAs could impede at various levels in the carcinogenesis, that is, blocking carcinogenesis initiation or suppressing cancer promotion, progression, angiogenesis, invasion and metastasis [2]. In carcinogenesis initiation, ChAs could alter the procarcinogens metabolisms, governed by phase I and phase II enzymes [3], by conjugation and excretion of the reactive and toxic metabolites [4].

On the one hand, phase I enzymes, for example, NADPH-cytochrome c reductase, Cyt b5 and Cyt P450 (CYP), participate in the reduction, oxidation or hydrolysis of xenobiotics producing end products that are mostly electrophilic species able to react with biomolecules, for example proteins and DNA, generating damages and cellular disruption that could promote the initiation of carcinogenic process. On the contrary, phase II enzymes, for example, NAD(P)H:quinone reductase (QR, EC 1.6.5.2), glutathione *S*-transferase (GST EC 2.5.1.18) and UDP-glucuronosyltransferase [5], participate in the conjugation of xenobiotics with endogenous molecules, for example glucuronic acid and glutathione, facilitating xenobiotic excretions reducing their carcinogenic properties. QR, regarded as a phase II enzyme due to its protective functions, is induced together with other phase II enzymes and regulated by enhancers like to those that censor GST regulation [6]. Increases in phase II enzymes levels, for example QR or GST, have been correlated with protection against carcinogenesis, induced by chemicals, in the initiation and promotion phases [1,2,6].

The ChAs that induce detoxification enzymes are classified as monofunctional and bifunctional inducers. ChAs belonging to the group of monofunctional inducers (green in Figure 1A) increase selectively phase II enzymes by activation of the antioxidant response element (ARE) via the Keap1-Nrf2 system. On the other hand, the bifunctional inducers (red in Figure 1a) increase both phase I and phase II enzymes by binding to the Ah receptor and then the Ah receptor-ligand complex is translocated to the nucleus, through the Ah nuclear translocator receptor (Arnt, Figure 1A), activating the xenobiotic response element.

Many compounds were found to induce genes of phase II enzymes [7,8,9,10,11]. Specifically, chalcones and hybrids Michael acceptor-dithiolethiones have been studied by our group. Chalcone **(I)** displayed the best behaviors in our *in vivo* tumorigenesis/chemopreventive and acute toxicity studies resulting monofunctional inducers (Figure 2A) [12,13], increasing liver phase II (QR and GST) enzyme activity and decreasing phase I (CYP1A1/CYP1A2, EC 1.14.14.1) activity [14]. Among the hybrid derivatives, compounds **(II–IV)** [15,16] resulted to be the greater monofunctional inducers of both phase II enzymes, QR and GST.

In this context and as a continuation of our previous work, we have undergone efforts in order to identify new chemotypes as monofunctional inducers by utilizing a small library of compounds from our laboratory. Observing the chemical structures of the previous ChAs **(III)** and **(IV)**, this library was focused taking into account the following structural criteria (Figure 2A): compounds with five member-ring and with heteroatom–heteroatom bond; compounds with α,β-unsaturated heterocarbonyl; and compounds with α,β-unsaturated carbonyl. Considering these three criteria, we selected compounds derived from the following moieties (Figure 2B): pyrazole derivative **(1)**, 1,2,4-oxadiazole derivative **(2)**, 1,2,5-oxadiazole derivative **(3)**, 1,2,3-thiadiazole derivative **(4)**, 1,2,4-thiazole derivative **(5)**, 1,3,4-oxathiazole derivative **(6)**; thienyl hydrazone derivative **(7)**, α,β-unsaturated-oxime derivative **(8)**, α,β-unsaturated *N*-oxide derivatives **(9)** and **(10)**, coumarin derivatives **(11–13)**; others α,β-unsaturated carbonyl derivatives **(14)** and **(15)**.

Herein, we describe the evaluation of selected compounds **(1–15)** (Table 1) as monofunctional enzymatic inducers measuring QR phase-II enzyme activity in an *in vitro* model using wild-type mouse hepatoma Hepa-1c1c7 and Arnt defective mutant BpRc1 cells [17,18]. Based on the results of this first analysis, we deepened the study analyzing new derivatives from those chemotypes with the best desired bioactive profile. Consequently, derivatives **(16–54)** (Tables 2–3) were studied in their capability to induce QR enzyme activity. From the best derivatives, since 1,2,4-triazine 4-oxide and tetrahydropyrimidinone scaffolds, studies on GST phase-II enzymes activities were performed. In these studies the same cell lines, wild-type mouse hepatoma Hepa-1c1c7 and Arnt defective mutant BpRc1 cells, were used. For the most relevant derivatives the concentrations required for doubling enzymatic activity (CDs), and chemopreventive indexes (CIs, ratio between IC_50_ and CDs) [16], were determined.

## Results & discussion

Thirteen different chemotypes which complied with the pre-established structural requirements were selected from our chemical library, namely: pyrazole, 1,2,4-oxadiazole, 1,2,5-oxadiazole, 1,2,3-thiadiazole, 1,2,4-thiazole and 1,3,4-oxathiazole, belonging to heteroatom–heteroatom bond containing five member-ring category; thienyl hydrazone, α,β-unsaturated oxime, benzimidazole 1,3-dioxide and 1,2,4-triazine 4-oxide, belonging to α,β-unsaturated heterocarbonyl category; coumarin, 4-carboxaldehydeimidazole and tetrahydropyrimidine, belonging to α,β-unsaturated carbonyl category. The selected derivatives, **(1–15)** (Figure 2), were prepared previously in our laboratory with different bioactivity [19–28].

The selected derivatives, **(1–15)**, were tested *in vitro* for their capabilities to induce exclusively phase II enzymes. A model that uses two different cellular systems was employed, wild-type Hepa-1c1c7 and mutant BpRc1 cells (Figure 1B). The increase in enzymatic activity in the mutant cell line concomitant with the increase in the wild type indicates that the compound tested is a monofunctional inducer (induces only phase II enzymes, Figure 1A). When the increase is only in the wild type, the compound is considered as a bifunctional inducer (induction of phase I and phase II enzymes, Figure 1A) [17]. According to this expected biological behavior we used as inducer descriptor the ratio of specific activity between wild and mutant cells, r_H__/B_. When the compound has a value of r_H__/B_ near to 1, and it increases the specific enzymatic activity, it displays a monofunctional behavior. However, when the compound has a value of r_H__/B_ higher than 1, and it increases the specific enzymatic activity, it displays a bifunctional behavior. As a primary screening, QR activities were determined in both cellular models using the compounds at 10 μM (Table 1). 4′-Bromoflavone (4-BFV) [11] was used as control finding that it displays the typical behavior of a bifunctional inducer (Table 1), that is, high QR expression in wild-type cells with an r_H__/B_ of 3.0.

This first series of compounds displayed a wide variety of results and, in general, no tendency of bioactivity was evidenced in the different chemotypes. Compounds **(2)**, **(4)** and **(11)-(14)** did not produce effect on the QR-specific activity displaying absence of induction in both cellular systems. The absence of coumarin **(11)** activity was very surprising, since there are reports [29,30] that the coumarins have the ability to induct phase II enzymes activity. Therefore we evaluated other coumarins, **(12)** and **(13)**, with different structural features finding the same bioresponse that compound **(11)**. The compounds **(1)**, **(5**) and **(10)** displayed a monofunctional profile (r_H__/B_ of 0.92, 1.13 and 1.08, respectively). Between them the best monofunctional inducer was the 1,2,4-triazine 4-oxide **(10)** because it is almost double the values of QR-specific activity, with respect to untreated cells, at the assayed conditions in both the tested cellular systems. The compounds **(3)** and **(15)** displayed an intermediate behavior as monofunctional and bifunctional agents (r_H__/B_ = 1.77 and 1.63, respectively). A particular biological behavior showed compounds **(6)** and **(7)**. They induced preferentially in the mutant cells, r_H__/B_ = 0.58 and 0.55 respectively, probably extra mechanisms were operative in this cell line that increased the QR activity. Finally, the α,β-unsaturated heterocarbonyls **(8)** and **(9)**, like the control compound, 4-BFV, were clearly bifunctionals displaying higher QR-specific activities on the wild-type cells (r_H__/B_ = 4.70 and 3.31, respectively).

In a second approach, we deepened the study analyzing new derivatives from the initially identified most characteristic chemotypes, namely: pyrazole, 1,2,5-oxadiazole, 1,2,4-thiadiazol, 1,2,4-triazine 4-oxide and tetrahydropyrimidine. Consequently, we selected new derivatives, from our chemolibrary [19,21,25,31–37], varying some moieties in these frameworks, that is, pyrazoles **(16)** and **(17)**, 1,2,5-oxadiazoles **(18)** and **(19)**, 1,2,4-thiadiazoles **(20)**, **(21)** and **(22)**, 1,2,4-triazine 4-oxides **(23)–(25)** and tetrahydropyrimidines **(26–28)** (Figure 3). We could state from the different chemotypes the following conclusions (Table 2): conversion of pyrazole to indazole made the chemotype as bifunctional, that is, indazole **(17)** (r_H__/B_ = 8.06); the rest of analyzed 1,2,5-oxadiazoles, like the compounds **(6)** and **(7)**, showed a particular biological behavior where the induction is preferential in the mutant cells, r_H__/B_ = 0.72 for 1,2,5-oxadiazol **(18)** and 0.65 for the benzo-derivative, **(19)**, probably extra mechanisms were operative in this cell that increased the QR activity in the mutant line BpRc1; unlike the compound **(5)** the new evaluated 1,2,4-thiadiazoles, **(20–22)**, displayed an intermediate profile between monofunctional and bifunctional behaviors (r_H__/B_ = 1.28, 1.03 and 1.47, respectively) having the 1,2,4-thiadiazol-5-one **(22)** the most bifunctional activity maybe as result of the structural change in the heterocycles system; derivatives of 1,2,4-triazine chemotype displayed very diverse behavior while compound **(23)** was clearly monofunctional the corresponding dioxidized analog, that is, **(24)**, was bifunctional and the simplest analog **(25)** was intermediate, between monofunctional and bifunctional, almost reaching three fold the values of QR-specific activity, with respect to untreated cells, at the assayed conditions in both cellular systems tested; derivatives belonging to tetrahydropyrimidine chemotype, that is, **(26–28)**, were monofunctional being carbonyl analogue **(28)** the best reaching the double of the values of QR-specific activity, respect to untreated cells, at the assayed conditions in both cellular systems tested.

The excellent results with derivatives of the families of 1,2,4-triazine 4-oxide and tetrahydropyrimidine led us to perform a third approach, it was deepened in the study of new structural modifications on these systems. Consequently, we selected new derivatives of the 1,2,4-triazine 4-oxide system modifying substituents at the 3- and 5-position of the heterocycle and the level of oxidation (derivatives [29–36], Table 3) [25,34,35]. With these results we could identify new 1,2,4-triazine 4-oxides with better activities than the parent compounds, **(10)**, **(23)** and **(25)**, being derivatives **(31)** and **(32)** the best monofunctional inductors of phase II enzymes in both cellular systems in the assayed conditions (r_H__/B_ = 1.01 and r_H__/B_ = 1.03, respectively). Additionally, derivative **(33)** displayed adequate activity showing a profile with greater QR induction on mutant cells. Particularly, **(31–33)** have at 5-position of the triazine heterocycles a substituent with electron-withdrawing moieties. Unlike derivative **(23)**, the electron-donor substituted derivatives, that is, **(29)** and **(30)**, displayed a clear bifunctional behaviors (r_H__/B_ = 1.93 and r_H__/B_ = 3.73, respectively). Furthermore, the dioxide derivative **(35)**, like the dioxide derivative **(24)** (Table 2), and the saturated derivative **(36)** were bifunctional inductors (r_H__/B_ = 4.96 and r_H__/B_ = 2.05, respectively). In reference to the new derivatives of the tetrahydropyrimidine system we selected compounds where 2-thiocarbonyl and 4-phenyl groups were varied (derivatives **(37–54)**, Table 4) [27,28,36,37]. Unlike the parent tetrahydropyrimidinone **(28)** some of the 2-carbonyl derivatives, that is, **(44)**, **(45)**, **(47)** and **(50)**, were poor QR inductors. However, derivatives **(46)** and **(49)** showed QR induction but with particular performances, clear induction on wild-type cells and enhanced induction on mutant ones (Table 3). Finally, the 2-carbonyl 4-fluoro-substituted derivative **(48)** displayed a clear bifunctional behavior. On the other hand, some of the 2-thiocarbonyl analogs, that is, **(38–40)**, were good monofunctional inductors with r_H__/B_ = 0.91, r_H__/B_ = 0.82 and r_H__/B_ = 0.75, respectively, however none of them was better than the parent compounds **(15)** and **(27)**. We could not identify a clear relationship between phenyl-substituent electronic characteristic and activity. Finally, the modification on the thiocarbonyl moiety producing thioether conducted to agents with different biological behaviors being derivatives **(52)** and **(53)** the most promising compounds improving the activity of the parent thioether **(26)** (Table 3).

In order to complete the study, for some of the most relevant identified mono- or partially monofunctional inducers, that is, **(3)**, **(15)**, **(23)**, **(25)**, **(26)**, **(28)**, **(31)**, **(32)**, **(49)**, **(52)** and **(53)**, the CI were determined (Table 5). The CI is defined as the ratio of the concentration which inhibited the growth of cell lines by 50% (IC_50_) and the concentration that double QR-specific activity in the same cell line (CD). Also, in this study 4-BFV and *t*-butylhydroquinone (*t*-BHQ) were included as controls. The CIs revealed that tetrahydropyrimidine **(28)** was the best potential cancer ChA with clear monofunctional inducer capacity. It possessed, in both cellular models, similar values of CDs and lower cytotoxicity. The compound **(3)**, with a monofunctional behavior, possessed, within the different studied compounds, the lowest values of CDs (for both cells, wild type and mutant). This pointed **(3)** as a good potential cancer ChA. In the group of 1,2,4-triazine 4-oxide with clear monofunctional behavior derivative **(23)** displayed the best CIs values. Derivative **(32)** with the best CDs values was toxic against wild-type cells yielding a very low value of CI in this system. On the other hand, the tetrahydropyrimidines **(26)** and **(49)** showed a discrete activity in both cellular systems. The 1,2,4-triazine 4-oxides **(25)** and **(31)** and the tetrahydropyrimidines **(15)**, **(52)** and **(53)** showed like the control compounds 4-BFV and *t-*BHQ, partial bifunctional behaviors with lower CD values on wild-type system.

In order to complete the information about the capability of the studied compounds to induce phase-II enzymes we also analyzed for the last compounds, that is, **(3)**, **(15)**, **(23)**, **(25)**, **(26)**, **(28)**, **(31)**, **(32)**, **(49)**, **(52)** and **(53)**, the modification on GST in both cellular models (Figure 4). Again, according to r_H__/B_ (defined for GST specific activity induction), the positive control, 4-BFV, was classified as bifunctional inducers displaying higher GST specific activity on the wild-type cells, that is, r_H__/B_ of 2.7. Again, the 1,2,4-triazine 4-oxide **(32)** was classified as monofunctional inducer (r_H__/B_ of 0.98) being the best GST inducer. Similarly the tetrahydropyrimidines **(28)**, **(15)**, **(52)** and **(53)** were monofunctional inducers, r_H__/B_ of 1.01, 0.96, 1.15 and 1.23, respectively, being the best GST inducer the 2-carbonyl derivative **(28)**. The 1,2,5-oxadiazole 2-oxide **(3)**, the 1,2,4-triazine 4-oxide **(25)** and the tetrahydropyrimidines **(26)** and **(49)** displayed intermediate monofunctional behavior, r_H__/B_ of 1.51, 1.37, 1.39 and 1.49, respectively, where **(3)** was the best GST inducer in this group of compounds. Triazines **(23)** and **(31)** showed particular behaviors in the GST induction where both compounds duplicate, respect to untreated cells, this phase II-activity in the mutant cell line without induction on the wild cells, probably extra mechanisms were operative in this cellular line that increased the GST activity.

In order to confirm that the mechanism of enzyme-II induction is via ARE activation [38], we evaluated the Nrf2 nuclear translocation ability, using the 1,2,5-oxadiazole **(3)** as the model compound. Therefore, to further investigate effects of **(3)** on the Nrf2/ARE activation, we examined the subcellular location of Nrf2 in Hepa-1c1c7 cells after 1,2,5-oxadiazole treatment. Immunofluorescence analyses (Figure 5) showed Nrf2 accumulation in the nucleus of cells upon treatment.

Lipinski's rule of five [39] serves as a guide to know the bioavailability of some studied compounds. Analysis of compounds **(3)**, **(23)**, **(28)**, **(32)** and **(49)** (Figure 6) suggested that it would be orally administered fitting, in all the cases, to the rule (Table 6).

## Experimental

### Chemistry

All the compounds were from our in-house chemical-library. They were previously synthesized [19–28,31–37].

### Biology

#### Cell culture & conditions

Mouse hepatoma Hepa-1c1c7 cell (ATCC, CRL-2026) and its mutant BpRc1 (ATCC, CRL-2217) were purchased from American Type Culture Collection (ATCC, Manassas, VA, USA). They were cultured in α-MEM and DMEM containing 10% FBS, respectively, and grown at 37°C under a 5% CO_2–_95% air atmosphere.

#### Preparation of cytosolic fraction & assay procedure

A suspension containing 8.0 × 10^4^ cells in 1.0 ml of milieu was seeded in 24-well plate and incubated 24 h to allow cell attachment. The milieu was aspirated and the cells washed twice with 1.0 ml of phosphate-buffered saline (PBS). Compounds were tested in triplicate at the desired concentration, for the initial study at 10 μM, for the CD (concentration required to double QR activity) determination at 2.5, 5.0, 8.0, 10.0 and 20.0 μM, in 1.0 ml of fresh milieu and not exceeding 0.5% v/v DMSO. The cells were treated for 48 h, the milieu was aspirated, the cells washed twice with PBS and then detached using 0.1 ml trypsin/EDTA. Fresh milieu was added and 1.0 ml of cellular suspension was transferred into 1.5 ml tubes and centrifuged at 10.000 g for 5 min. The milieu was removed, cells rinsed once with 0.5 ml PBS and centrifuged again in the same conditions. PBS was removed and the cell pellet was suspended in 0.5 ml 25mM Tris-HCl buffer at pH 7.4. The suspension was lysed by sonication for 10 s on ice. The lysed suspension was centrifuged at 10.000 g for 5 min. The supernatant (cytosolic fraction) was used to measure QR or GST activity.

#### Determination of QR activity

Using 2,6-dichloroindophenol (2,6-DCIP) as the substrate, the cytosolic QR activity was measured as the decrease in absorbance at 600 nm, due to the reduction of 2,6-DCIP by QR. The assay buffer consisted of 25 mM Tris-HCl (pH 7.4), 5 μM FAD, 0.2 mM NADH, 40 μM 2,6-DCIP, 6 mg/100 ml bovine serum albumin and 10 μL/100 mL Tween-20. 5 μg of total protein for each sample was added to the cuvette containing 1 ml of assay buffer. The QR activity was determined, at room temperature, by the decrease in absorbance per min per mg of the total protein of the sample. The time of the determination was 1 minute.

#### Determination of GST activity

Using 1-chloro-2,4-dinitrobenzene (CDNB, 1mM) and GSH (1 mM) as the substrate, cytosolic GST activity was measured as the increase in absorbance at 340 nm, due to the formation of GS-CDNB adduct by GST. The assay buffer consisted of Na_2_HPO_4_/NaH_2_PO_4_ (pH 6.5). Five μg of total protein for each sample was added to the cuvette containing 1 ml of assay buffer. GST activity was determined, at room temperature, by the increase in absorbance per min per mg of the total protein of the sample. The time of the determination was one minute.

#### Cytotoxicity assay

Cells were seeded in 96-well plates (20 × 10^3^ cells/well) in 100 µl final volume and were allowed to grow 24 h. After that, 100 µl of each concentration, 10.0, 50.0, 100.0, 150.0 and 200.0 µM, of the tested compounds in the culture milieu were added onto the cells and were further incubated for 48 h. Then, cells were fixed with ice-cold trichlorocetic acid for 1 h at 4ºC, the plates were washed five-times in distilled water and allowed to dry in the air. Sulphorhodamine solution (SRB, 50 µl) was added to each well of the dry 96-well plates and allowed staining at room temperature for 30 min. The SRB solution and unbound dye were removed by washing the plates quickly with 1% v/v acetic acid, five times. The washed plates were dried in the air. The bound SRB was solubilized by adding 100 µl of 10 mM unbuffered Tris Base (pH 10.5) to each well and shaking for 5 min on a shaker platform. The developed color was read in a 96-well plate reader at 492 nm. The optical density of SRB in each well is directly proportional to the cell number consequently the optical density values were plotted against concentration and the IC_50_ value were determined.

#### Immunofluorescence staining

Hepa-1c1c7 cells were grown in 6-well plate containing glass cover slips to 60% confluence and then treated with 10 or 20 μM of compound **(3)** during 6 h. Untreated cells were included as a control. Culture milieu was removed, cells were fixed by adding 4% paraformaldehyde solution and incubated at room temperature for 10 min. Cells were permeabilized with PBS containing 0.1% Triton X-100. The cells were then blocked with 10% (v/v) goat serum for 45 min and incubated with mouse antibody against Nrf2 overnight at 4°C. Cover slips were washed and incubated in the dark with Alexa Fluor 594-conjugated secondary antibody (dilution 1:1,000) at room temperature for 1 h. The nuclei were co-labeled with DAPI solution. Finally, the cover slips were washed and mounted on cover slides using ProLong^®^ Gold Antifade Reagent. The images were collected using a laser confocal microscope Leica TCS SP5 equipped with a 63× oil objective 1.4NA. Images were processed using LASAF 2.7.3v software.

## Conclusion & future perspective

We reported the identification of new chemotypes, from a focused library, with ability to induce phase II enzymes. The focuses of the chemolibrary were compounds with heteroatom–heteroatom bond in five member-rings, compounds with α,β-unsaturated heterocarbonyl and compounds with α,β-unsaturated carbonyl. Among the analyzed compounds, 1,2,5-oxadiazole **(3)**, 1,2,4-triazine **(23)** and **(32)** and tetrahydropyrimidine **(28)** and **(49)** (Figure 6) resulted to be the great monofunctional inducers of both phase II enzymes, QR and GST. The induction property of compound **(3)** could be justified by its capacity to release nitric oxide, NO [40]. Recently, it has been reported that some NO-releasing compounds could act as monofunctional inducers of phase II enzyme through *S*-nitrosylation of 273-Cys and/or 288-Cys of Keap1 or *S-*guanylation of 434-Cys of Keap1, from 8-nitro-cGMP generated by NO-nitration of cGMP, and subsequent translocation to the nucleus of Nrf-2 [41–44]. Additionally, the high electrophilicity of carbon 3 of the 1,2,5-oxadiazole **(3)** [45,46] could promote the direct covalent bonding to Keap1 promoting its structural changes and the concomitant Nrf-2 nuclear translocation. On the other hand, compounds like **(23)**, **(32)**, **(29)** and **(49)** have been described as anticancer agents [25–28,34–37] that transform them as agents with potential dual use, chemopreventive and antitumor drugs.

For the abovementioned points these compounds are good candidates for further *in vivo* studies of cancer chemopreventive activity.

**Table 1.** **Induction of quinone reductase activity on Hepa-1c1c7 and BpRc1 of the studied compounds, evaluated at 10 μM dose.**

**Compound**	**QR activity^†^**		
	**Hepa-1c1c7**	**BpRc1**	**r_H/B_^‡^**	
**(1)**	1.22±0.40	1.32±0.46	0.92
**(2)**	1.15±0.46	1.18±0.31	0.97
**(3)**	4.20±0.02	2.37±0.30	1.77
**(4)**	1.22±0.03	0.91±0.02	1.34
**(5)**	1.49±0.50	1.32±0.40	1.13
**(6)**	0.98±0.55	1.70±0.64	0.58
-	-	-	-
-	-	-	-
-	-	-	-
4-BFV^§^	4.45±0.35	1.49±0.56	3.0
**(7)**	0.99±0.05	1.80±0.40	0.55
**(8)**	4.70±1.11	1.00±0.09	4.70
**(9)**	2.78±1.10	0.84±0.13	3.31
**(10)**	1.83±0.19	1.70±0.20	1.08
-	-	-	-
**(11)**	1.02±0.10	0.97±0.08	1.05
**(12)**	1.12±0.10	0.96±0.07	1.17
**13)**	0.97±0.02	1.15±0.18	0.84
**(14)**	0.98±0.39	0.96±0.30	1.02
**(15)**	2.51±0.01	1.54±0.02	1.63

^†^Ratio of QR-specific activity= QR-specific activity of treated cells/QR-specific activity of control cells. QR-specific activity of control cells: for Hepa-1c1c7 line = 0.1400 ± 0.0013 U/mg protein, for BpRc1 line = 0.2200 ± 0.0063 U/mg protein.

^‡^r_H/B_: ratio QR-specific activity on Hepa-1c1c7/on BpRc1.

^§^4-BFV: 4′-bromoflavone; QR: Quinone reductase.

**Table 2.** **Induction of quinone reductase activity on Hepa-1c1c7 and BpRc1 of the second group of compounds, from the most active chemotypes, evaluated at 10 μM dose.**

**Family of compound**	**Compound**	**QR activity^†^**			**Family of compound**	**Compound**	**QR activity^†^**		
		**Hepa-1c1c7**	**BpRc1**	**r_H/B_^‡^**			**Hepa-1c1c7**	**BpRc1**	**r_H/B_^‡^**
	**(16)**	1.43±0.30	1.57±0.16	0.91		**(23)**	1.62±0.13	1.85±0.22	0.88
Pyrazoles	**(17)**	9.51±2.07	1.18±0.74	8.06	Triazines	**(24)**	1.48±0.07	0.39±0.01	3.79
						**(25)**	3.54±0.64	2.52±0.33	1.40
	**(18)**	0.98±0.30	1.36±0.43	0.72		**(26)**	1.38±0.06	1.19±0.02	1.16
Oxadiazoles	**(19)**	1.10±0.03	1.70±0.10	0.65	Tetrahydropyrimidines	**(27)**	1.47±0.11	2.09±0.06	0.70
						**(28)**	2.19±0.03	3.29±0.01	0.67
	**(20)**	1.99±0.20	1.55±0.09	1.28					
Thiadiazoles	**(21)**	1.21±0.11	1.17±0.11	1.03					
	**(22)**	1.38±0.70	0.94±0.66	1.47					

^†^Ratio of QR-specific activity= QR-specific activity of treated cells/QR-specific activity of control cells. QR-specific activity of control cells: for Hepa-1c1c7 line = 0.1400 ± 0.0013 U/mg protein, for BpRc1 line = 0.2200 ± 0.0063 U/mg protein.

^‡^r_H/B_: ratio QR-specific activity on Hepa-1c1c7/on BpRc1.

QR: Quinone reductase.

**Table 3.** **Induction of quinone reductase activity on Hepa-1c1c7 and BpRc1 of the third group of studied 1,2,4-triazine 4-oxides, evaluated at 10 μM dose.**


**Compound**	**n**	**Ar**	**QR activity^†^**		
			**Hepa-1c1c7**	**BpRc1**	**r_H/B_^‡^**
**(29)**		4-(dimethylamino)phenyl	2.08 ±0.21	1.08 ±0.17	1.93
**(30)**		Benzo[*d*][1,3]dioxol-5-yl	3.10 ±0.14	0.83 ±0.02	3.73
**(31)**	0	4-chlorophenyl	2.13 ±0.09	2.10 ±0.06	1.01
**(32)**		5-nitro-2-thienyl	4.29 ±0.01	4.15 ±0.82	1.03
**(33)**		5-nitro-2-furyl	1.34 ±0.07	2.41 ±0.03	0.56
**(34)**	1	4-(dimethylamino)phenyl	0.91 ±0.04	1.15 ±0.02	0.79
**(35)**			1.34 ±0.62	0.27 ±0.03	4.96
**(36)**			2.32 ±0.01	1.13 ±0.02	2.05

^†^Ratio of QR-specific activity= QR-specific activity of treated cells/QR-specific activity of control cells. QR-specific activity of control cells: for Hepa-1c1c7 line = 0.1400 ± 0.0013 U/mg protein, for BpRc1 line = 0.2200 ± 0.0063 U/mg protein.

^‡^r_H__/B_: ratio QR-specific activity on Hepa-1c1c7/on BpRc1.

QR: Quinone reductase.

**Table 4.** **Induction of quinone reductase activity on Hepa-1c1c7 and BpRc1 of the third group of studied tetrahydropyrimidines, evaluated at 10 μM dose.**

	
**-R**	**Compound**	**QR activity^†^**				**Compound**	**QR activity^†^**		
		**Hepa-1c1c7**	**BpRc1**	**r_H/B_^‡^**			**Hepa-1c1c7**	**BpRc1**	**r_H/B_^‡^**
-4-NMe_2_	**(37)**	1.63 ±0.50	1.14 ±0.06	1.43		**(44)**	1.02 ±0.02	1.79 ±0.01	0.57
-3-OH	**(38)**	1.33 ±0.01	1.46 ±0.06	0.91		**(45)**	1.14 ±0.23	1.01 ±0.17	1.13
-3,4-(OCH_2_O)	**(39)**	1.41 ±0.07	1.72 ±0.01	0.82		**(46)**	1.69 ±0.09	2.93 ±0.48	0.58
-3-OMe	**(40)**	1.23 ±0.11	1.63 ±0.05	0.75		**(47)**	1.17 ±0.02	0.96 ±0.13	1.22
-4-F	**(41)**	2.27 ±0.02	1.28 ±0.02	1.77		**(48)**	4.18 ±0.32	1.74 ±0.02	2.40
-3-F	**(42)**	1.17 ±0.02	1.20 ±0.01	0.98		–	–	–	–
-4-CN	**(43)**	1.93 ±0.09	1.26 ±0.03	1.53		**(49)**	1.50 ±0.37	2.19 ±0.28	0.68
-3-NO_2_	–	–	–	–		**(50)**	1.14 ±0.12	0.98 ±0.01	1.16

**-R/-R**^†^	**Compound**	**Hepa-1c1c7**	**BpRc1**	**r_H/B_^‡^**	**-R/-R’**	**Compound**	**Hepa-1c1c7**	**BpRc1**	**r_H/B_^‡^**
-3-OH/-Et	**(51)**	1.68 ±0.03	0.96 ±0.04	1.75	-3-NO_2_/-Et	**(53)**	1.54 ±0.03	1.69 ±0.01	0.91
-3-F/-Et	**(52)**	2.63 ±0.04	1.99 ±0.01	1.32	3-NO_2_/-Bz^c^	**(54)**	0.23 ±0.01	1.77 ±0.12	0.13

^†^Ratio of QR-specific activity= QR-specific activity of treated cells/QR-specific activity of control cells. QR-specific activity of control cells: for Hepa-1c1c7 line = 0.1400 ± 0.0013 U/mg protein, for BpRc1 line = 0.2200 ± 0.0063 U/mg protein.

^‡^r_H/B_: ratio QR-specific activity on Hepa-1c1c7/on BpRc1. ^c^Bz= benzyl.

Bz: benzyl; QR: Quinone reductase.

**Table 5.** **Effects of selected compounds (3), (15), (23), (25), (26), (28), (31), (32), (49), (52) and (53), on Hepa-1c1c7 and BpRc1 cytotoxic effects (IC_50_), induction of quinone reductase activity and chemopreventive indexes.**

**Chemotype**	**Compound**	**Hepa-1c1c7^†^**			**BpRc1^†^**		
		**IC_50_ (μM)**	**CD^‡^ (μM)**	**CI^§^**	**IC_50_ (μM)**	**CD^‡^ (μM)**	**CI^c^**
1,2,5-oxadiazole*N*-oxide	**(3)**	42.0	4.4	9.5	52.0	5.4	9.6
1,2,4-triazine 4-oxide	**(23)**	136.0	18.0	8.0	83.0	20.0	4.2
	**(25)**	>200.0^#^	4.4	>46.0	>200.0^#^	14.0	>14.0
	**(31)**	4.0	20.0	0.2	<1.0	>20.0	<0.05
	**(32)**	2.0	7.5	0.3	108.0	5.0	22.0
Tetrahydropyrimidine	**(15)**	>200.0^#^	2.8	>70.0	>200.0^#^	14.0	>14.0
	**(26)**	68.2	12.0	5.7	122.0	20.0	6.1
	**(28)**	>200.0^#^	10.0	>20.0	>200.0^#^	6.2	>32.0
	**(49)**	>200.0^#^	>20.0	˜10.0	>200.0^#^	11.0	>19.0
	**(52)**	133.0	9.9	13.0	58.9	>20.0	<3.0
	**(53)**	67.0	>20.0	<3.4	151.0	>20.0	<7.6
	4-BFV	85.0	0.79	107	150.0	80.0	1.9
	*t-*BHQ	147.0	1.0	147	60.0	6.0	10.0

^†^Results are means of three independent experiments with standard deviation less than 10% in all cases.

^‡^CD: concentration required to double the quinone reductase specific activity.

^§^CI: ratio between IC_50_ and CD.

^#^Solubility problems in the biological milieu did not allow to probe higher doses.

*t-*BHQ: *t*-butylhydroquinone.

**Table 6.** **Lipinski's physicochemical properties for selected compounds.**

**Chemotype**	**Compound**	**Lipinski descriptors**				**Number of violation**	**Fits the rule**
		**MilogP^†^**	**MW**	**nON**	**nOHNH**		
1,2,5-oxadiazole	**(3)**	2.06	190.2	5	0	0	Yes
1,2,4-triazine	**(23)**	0.96	243.3	5	0	0	Yes
	**(32)**	0.71	264.3	7	0	0	Yes
Tetrahydropyrimidine	**(28)**	2.37	260.3	5	2	0	Yes
	**(49)**	2.13	285.3	6	2	0	Yes

^†^-miLogP was calculated usingMolinspiration online property calculation toolkit: [47].

**Executive summary**

Cancer chemoprevention is the prevention, delay or reversal of the carcinogenic process by administration of cancer chemopreventive agents.
We have studied compounds from our chemical-library with some structural frameworks.
Some compounds are good selective inducers of the phase II enzymes, quinone reductase and glutation *S*-transferase.
The best derivatives act as monofunctional inducers with excellent *in vitro* chemopreventive indexes.
